# Overnight wakefulness impairs next-day postprandial glucose in young women independent of sex hormones

**DOI:** 10.1210/clinem/dgag038

**Published:** 2026-01-31

**Authors:** Pei Xue, Elisa M S Meth, Samira F M Noory, Viviana Rossi, Dina Motoshaleh, André P Pacheco, Heike Vogel, Diana A Nôga, Christian Benedict

**Affiliations:** Department of Pharmaceutical Biosciences, Uppsala University, Uppsala 751 24, Sweden; Department of Pharmaceutical Biosciences, Uppsala University, Uppsala 751 24, Sweden; Department of Pharmaceutical Biosciences, Uppsala University, Uppsala 751 24, Sweden; Department of Pharmaceutical Biosciences, Uppsala University, Uppsala 751 24, Sweden; Department of Pharmaceutical Biosciences, Uppsala University, Uppsala 751 24, Sweden; Department of Pharmaceutical Biosciences, Uppsala University, Uppsala 751 24, Sweden; Department of Research and Innovation, Division of Mental Health and Addiction, Oslo University Hospital, Oslo 037 2, Norway; Institute of Clinical Medicine, Faculty of Medicine, University of Oslo, Oslo 031 5, Norway; German Center for Diabetes Research (DZD e.V.), München-Neuherberg 857 64, Munich, Germany; Research Group Nutrigenomics of Obesity and Department of Experimental Diabetology, German Institute of Human Nutrition Potsdam-Rehbruecke, Nuthetal 145 58, Germany; Department of Pharmaceutical Biosciences, Uppsala University, Uppsala 751 24, Sweden; Department of Pharmaceutical Biosciences, Uppsala University, Uppsala 751 24, Sweden

**Keywords:** night shift, postprandial glucose metabolism, indirect calorimetry, female sex hormones

## Abstract

**Context:**

Estradiol and progesterone may influence glucose regulation, but it remains unclear whether ovarian hormone levels modulate night shift–induced impairments in glucose metabolism.

**Objective:**

To investigate the effects of a night shift–like schedule on next-morning fasting and postprandial glucose metabolism in reproductive-aged women.

**Methods:**

Fifty-two women with an HbA1c <5.7% completed a protocol consisting of one night of sleep and one night of wakefulness in a laboratory environment. After each night, fasting blood samples were collected to measure estradiol, progesterone, insulin, leptin, and adiponectin. Insulin resistance was estimated using HOMA-IR, and the leptin-to-adiponectin ratio (LAR)—a marker of fasting and postprandial insulin sensitivity—was calculated. Morning postprandial glucose responses were assessed using an oral glucose tolerance test (OGTT), and substrate utilization was measured via respiratory quotient (RQ) using indirect calorimetry. Linear mixed-effects models were used to test the effects of condition and the progesterone-to-estradiol (P/E) ratio on all outcomes.

**Results:**

Fasting glucose, HOMA-IR, and LAR did not differ between conditions. Post-OGTT blood glucose at 60 minutes (mean estimated difference: 0.58 mmol/L, *P* = .016) and peak glucose (0.42 mmol/L, *P* = .037) were higher following the night shift compared with sleep. The P/E ratio was not significantly associated with fasting or postprandial glucose. Pre-OGTT RQ was lower after the night shift (−0.029, *P* < .001), and adjusting for pre-OGTT RQ attenuated the post-OGTT glucose differences between conditions (*P* ≥ .097).

**Conclusion:**

One night of wakefulness—as occurs in night shift work—impaired next-morning glucose tolerance in women, independent of ovarian hormone variation. Accordingly, choosing low–glycemic index foods for the first meal after a night shift may help reduce postprandial glucose excursions.

Night shift work is a cornerstone of modern society, supporting essential services in healthcare, industry, and public safety. However, working at night requires remaining awake during a period when the human body is biologically programmed to rest and recover, which may pose health risks (1). Consistent with this, epidemiological studies have consistently reported an increased risk of prediabetes and type 2 diabetes among night shift workers (2-5). Controlled human studies have further shown that experimental paradigms inducing misalignment between sleep/wake cycles and endogenous circadian rhythms—as occurs during night shift work—impair blood glucose regulation (6-10).

Among women, glucose regulation may also be influenced by fluctuations in endogenous sex hormones. Estradiol, primarily secreted by the ovaries during the follicular and ovulatory phases, promotes glucose disposal in part by upregulating skeletal muscle glucose transporters (11, 12). In contrast, progesterone, which is primarily secreted during the luteal phase, has been reported to impair glucose homeostasis (13). For example, the use of a progesterone receptor antagonist reduced blood glucose levels and increased insulin secretion in mouse models (14). Whether inter-individual differences in the progesterone-to-estradiol (P/E) ratio—a measure reflecting ovarian hormone dynamics across the menstrual cycle—modulate the potential adverse effects of night shifts on fasting and postprandial glucose metabolism in women remains unclear.

Thus, in the present in-laboratory study, reproductive-aged women with normal HbA1c underwent one night of habitual sleep followed by one night of wakefulness (hereafter referred to as the experimental night shift), simulating an ecologically relevant scenario of night shift workers transitioning from an off- to an on-night shift period. We hypothesized that the experimental night shift, compared with the preceding night of sleep, would increase homeostatic model assessment of insulin resistance (HOMA-IR) values, indicative of systemic insulin resistance under fasting conditions (15), which we tested through blood sampling. We also assessed blood glucose responses during a standardized oral glucose tolerance test (OGTT) to determine whether typical OGTT metrics—such as postprandial and peak glucose levels—were exacerbated following the experimental night shift. To complement these measurements, we additionally determined the fasting leptin-to-adiponectin ratio (LAR), a marker previously linked to both fasting and postprandial insulin sensitivity, with higher values indicative of reduced fasting and postprandial insulin sensitivity (16). By examining the P/E ratio after experimental nights but before the OGTT, we aimed to investigate whether inter-individual differences in this ratio predicted glucose-related outcomes. We hypothesized that any adverse effects of the experimental night shift on HOMA-IR, LAR, and postprandial glucose would be attenuated in individuals with a ratio skewed toward estradiol, which has been shown to promote glucose disposal in skeletal muscle and other tissues (11, 12).

Finally, we used indirect calorimetry to determine the respiratory quotient (RQ), defined as the ratio of exhaled CO_2_ to inhaled O_2_. The RQ reflects the relative contribution of carbohydrates vs fats to energy metabolism, with values closer to 1.0 (under sedentary conditions) indicating predominant carbohydrate oxidation and values closer to 0.7 indicating predominant fat oxidation (17). We hypothesized that RQ would be lower after the experimental night shift compared to the sleep night, consistent with prior studies showing that overnight wakefulness increases reliance on fat and ketone oxidation (18) and nocturnal energy expenditure (19). Additionally, we hypothesized that lower morning RQ would be associated with higher post-OGTT glucose concentrations, as a slower or less efficient metabolic shift toward glucose oxidation following glucose ingestion may delay glucose disposal, leading to higher early postprandial blood glucose levels.

## Materials and methods

### Participants

A total of 673 women responded to study advertisements posted on campus and through social media. Of the 288 who completed the online screening questionnaire, 60 met all eligibility criteria: age 18 years or older; no history of physical, psychiatric, or sleep-related disorders; habitual sleep onset before 2200 hours or after 0000 hours; no night shift work; no use of hormonal contraceptives or other medications; consumption of fewer than six standard units of alcohol or caffeinated beverages per day; no drug or nicotine use; regular menstrual cycles (26-35 days); no premenstrual disorders; and no planned travel across time zones during the study period.

All study procedures adhered to the Declaration of Helsinki and were approved by the Regional Ethical Review Board in Uppsala, Sweden (DNR 66-2021/3.1). Written informed consent was obtained from all participants, who received monetary compensation. The protocol was preregistered on the Open Science Framework (OSF: https://osf.io/hq2mj/; 17 February 2022) and later registered on ClinicalTrials.gov (NCT06683248; 15 October 2024) to enhance transparency.

### Experimental scheme

**Figure 1:**
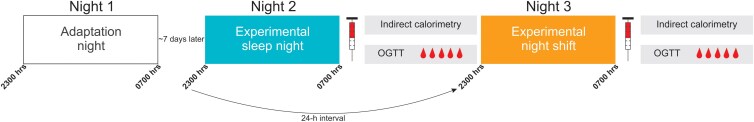
Experimental scheme. Schematic overview of the study design, illustrating the timeline of experimental conditions, interventions, and measurements. The blood-filled syringe icon represents fasting venous blood collection for the assessment of estradiol, progesterone, insulin, adiponectin, and leptin (∼0730 hours). The blood-drop icons indicate repeated capillary blood glucose measurements before and during the OGTT. Abbreviation: OGTT, oral glucose tolerance test.

Each participant completed both experimental conditions in a within-subject design, serving as her own control; no independent randomization between groups was performed. As illustrated in Fig. 1, women arrived at the sleep laboratory at approximately 2000 hours on the evening preceding the scheduled experimental night shift (representing their final night of regular sleep). Upon arrival, they received a standardized dinner (∼500 kcal; l; ∼65 g carbohydrate, ∼20 g fat, ∼20 g protein) and prepared for the sleep condition, scheduled between 2300 hours and 0700 hours. Sleep was monitored using the Dreem headband, a wearable reduced-montage electroencephalography device that has demonstrated good agreement with polysomnography-based assessments of total sleep duration (20).

The following morning, approximately 30 minutes after awakening (∼0730 hours), venous blood samples were collected for the assessment of estradiol, progesterone, insulin, adiponectin, and leptin. At approximately 0900 hours, participants underwent an oral OGTT, which required ingesting 75 g of dextrose dissolved in 300 mL of water within five minutes. Blood samples were obtained via finger lancet immediately before glucose ingestion (pre-OGTT) and at 30, 60, 90, and 120 minutes post-ingestion (post-OGTT). Blood glucose concentrations were analyzed using the Accu-Chek Mobile glucose meter (Roche Diagnostics, Basel, Switzerland), which meets the ISO 15197 accuracy standard within the normoglycemic and hyperglycemic ranges (21). Following each OGTT-related blood collection, RQ was measured using indirect calorimetry with the COSMED K5 system (COSMED, Rome, Italy) (22). The K5 was calibrated for flow and gas analyzers according to the manufacturer's recommendations. A Hans Rudolph face mask (Hans Rudolph Inc., Kansas City, Missouri, USA) was used to collect expired gases throughout each measurement. Oxygen consumption (VO_2_, mL/min) and carbon dioxide production (VCO_2_, mL/min) were recorded over 10 consecutive min, and RQ was calculated as VCO_2_ divided by VO_2_. RQ values closer to 0.7 indicate greater reliance on fat oxidation, whereas values approaching 1.0 reflect increased carbohydrate utilization (17). During the entire OGTT period, participants remained still, relaxed, and in a sitting position while breathing normally to minimize residual effects of incidental physical activity. Environmental conditions were tightly controlled, with room temperature maintained at 22 °C and noise kept to a minimum.

After the morning session, participants were permitted to leave the laboratory but were instructed not to sleep during the day. Compliance was verified using Fitbit activity monitors (Fitbit Inc., San Francisco, California, USA). Participants were also instructed to abstain from caffeinated beverages, to consume lunch as their final meal of the day, and to refrain from strenuous physical activity. At 2000 hours, participants returned to the sleep laboratory for the experimental night shift condition, which began with a standardized dinner identical to the one provided before the experimental sleep night. They were kept awake under continuous supervision and engaged in sedentary activities, such as reading, watching movies, or playing board games, in a room illuminated at ∼180 lux. No food consumption was permitted during this period to ensure that any differences in morning OGTT responses between the experimental night shift and sleep conditions were not confounded by priming effects from nocturnal eating during the night shift condition. The following morning, fasting blood samples were collected for hormone analyses, RQ was measured, and participants underwent the OGTT protocol as described above.

### Hormonal assays

Blood samples were collected using gold-top serum separator tubes containing a clot activator and polymer gel. Estradiol (E) and progesterone (P) concentrations were quantified using electrochemiluminescence immunoassay on the Roche Cobas Pro platform. The P/E ratio (pmol/pmol) was log-transformed and offset by +2 to prevent negative values when the ratio was below 1. Because neither blood estradiol nor progesterone exhibits diurnal variation in women, a single morning sample provides a valid assessment of menstrual-cycle phase (23).

Serum insulin was quantified using a commercially available ELISA kit (Insulin ELISA, Mercodia AB; RRID:AB_2877672). Serum leptin and total adiponectin concentrations were measured using the Human Leptin Quantikine ELISA kit (DLP00; Bio-Techne; RRID:AB_2783014) and the Human Total Adiponectin/Acrp30 Quantikine ELISA kit (DRP300; R&D Systems, Bio-Techne), respectively. No RRID is currently available for the DRP300 adiponectin assay. For statistical analyses, values below the detection limit for adiponectin (3.9 ng/mL; n = 4 in the sleep condition and n = 4 in the experimental night shift) were imputed using this lower limit. It should be noted that only total adiponectin was measured, rather than high–molecular-weight isoforms.

### Statistical analyses

All analyses were performed using SPSS version 30.0 (IBM Corp., Armonk, NY, USA). Data are reported as mean ± SD unless otherwise noted. Linear mixed-effects models (LMMs) were used to estimate parameter effects (β) and their 95% confidence intervals (95%-CI). LMMs were specified as an unstructured covariance matrix to account for within-subject correlations. Participant ID was included as a random intercept in all models to account for repeated measures. Model assumptions were assessed using standardized residual plots and Q–Q plots.

For fasting outcomes, HOMA-IR ([fasting insulin × fasting glucose]/22.5; ref. 15) and LAR (ng/µg) were analyzed in separate LMMs. We first tested potential interactions between condition and P/E ratio for each fasting outcome, while retaining the main effects. Non-significant interactions were removed, and simplified main-effect models were reported.

For the postprandial blood glucose analysis (ie, post-OGTT time points at 30, 60, 90, and 120 minutes), all models were adjusted for baseline fasting glucose. We first tested potential two-way interactions among condition, P/E ratio, and time point (one interaction term at a time), while retaining all relevant main effects. If an interaction was non-significant, the model was simplified to include main effects only. Peak postprandial glucose concentration (ie, the highest glucose value observed across the post-OGTT time points) was examined in a separate LMM, also adjusted for baseline fasting glucose. In this model, a condition × P/E ratio interaction was tested first; if non-significant, the final model included main effects only.

To assess whether differences in substrate utilization between the experimental night shift and sleep conditions could explain potential differences in postprandial blood glucose, we analyzed RQ values both before and during the OGTT. We then ran LMMs, including RQ values that differed significantly between conditions as an additional covariate. Overall, statistical significance was set at *P* < .05.

## Results

### Analytical samples and participant flow

Of the 60 women who met all eligibility criteria, two withdrew prior to the adaptation night, one was excluded due to obstructive sleep apnea detected during the adaptation night, and three chose not to continue thereafter. An additional two participants did not complete the OGTT following the experimental sleep night and subsequently withdrew. Consequently, 52 women completed the experimental sleep night, including RQ measurements and capillary blood glucose assessments the following morning. Fasting blood samples for estradiol, progesterone, insulin, leptin, and adiponectin were available for 49 women after a night's sleep. During the subsequent experimental night shift condition, three additional women discontinued participation, leaving 49 participants. All remaining participants had RQ measurements. One participant declined capillary blood glucose sampling entirely, and another refrained from glucose measurements after ingesting the oral glucose solution. Fasting blood samples for estradiol, progesterone, insulin, leptin, and adiponectin were available for 46 participants following the night shift condition.

### Cohort characteristics

Our cohort had a mean age of 25 ± 3 years and was predominantly of White ethnicity (White: 88.5%; Latin American: 5.8%; Asian: 1.9%; African: 1.9%; Mixed: 1.9%). Participants had a mean BMI of 22 ± 1.8 kg/m², and most had attained a university education (76.9%). HbA1c values were within the normal range (<5.7%), with a mean of 5.1 ± 0.2%. During the experimental sleep night, total sleep duration averaged 7.25 ± 0.57 hours.

Across sessions, blood estradiol and progesterone levels were within physiologically expected ranges (estradiol: 40-1177 pmol/L; progesterone: 0.2-67 nmol/L). Hormone concentrations were similar between conditions, with estradiol at 385 ± 261 pmol/L after sleep and 395 ± 237 pmol/L after the experimental night shift, and progesterone at 11.2 ± 15.8 nmol/L and 11.4 ± 16.2 nmol/L, respectively (*P* = .581 for estradiol and *P* = .881 for progesterone, respectively; derived from LMMs including condition as fixed effects and subject as a random factor). The resulting P/E ratio ranged from 1.82 to 3.98 (after sleep: 3.15 ± 0.50; after experimental night shift: 2.96 ± 0.67). Similarly, other metabolic indicators displayed substantial inter-individual variability (HOMA-IR: 0.27 to 2.88; LAR: 0.24 to 5037).

### HOMA-IR

Fasting blood glucose was similar between conditions, averaging 4.87 ± 0.51 mmol/L after sleep and 4.88 ± 0.54 mmol/L following the experimental night shift (*P* = .835; LMMs with condition as fixed effects and subject as a random factor). Fasting insulin levels were also comparable (sleep: 34.3 ± 12.5 pmol/L; night shift: 34.4 ± 14.4 pmol/L; *P* = .985).

In models including the multiplicative interaction term, there was no evidence of a condition × P/E ratio interaction on HOMA-IR (β [95% CI]: −0.108 [−0.46 to 0.24], *P* = .536). In models including only main effects, neither P/E ratio (β [95% CI]: 0.169 [−0.01 to 0.35], *P* = .067) nor condition (HOMA-IR, night shift: 1.30 [1.13-1.46] vs sleep: 1.22 [1.08-1.36], *P* = .500) significantly predicted HOMA-IR.

### LAR

Leptin concentrations were slightly higher following the experimental night shift (12.31 ± 7.08 ng/mL) than after sleep (11.64 ± 6.99 ng/mL; *P* = .057 for condition; derived from LMMs including condition as fixed effects and subject as a random factor), whereas adiponectin levels were virtually unchanged between sessions (experimental night shift vs sleep: 7.79 ± 4.46 vs 7.74 ± 4.30 µg/mL; *P* = .525 for condition).

In models including the multiplicative interaction term, the condition × P/E ratio interaction for LAR was not significant (β [95% CI]: 7.19 [−58.24 to 72.63], *P* = .825). In the main-effects model, LAR did not differ meaningfully between the experimental night shift and sleep conditions (β [95% CI]: 252.84 [−12.62 to 518.30] vs 240.88 [−31.52 to 513.28], *P* = .526), nor was it associated with the P/E ratio (β [95% CI]: 34.19 [−54.02 to 122.39], *P* = .439).

### OGTT

**Figure 2:**
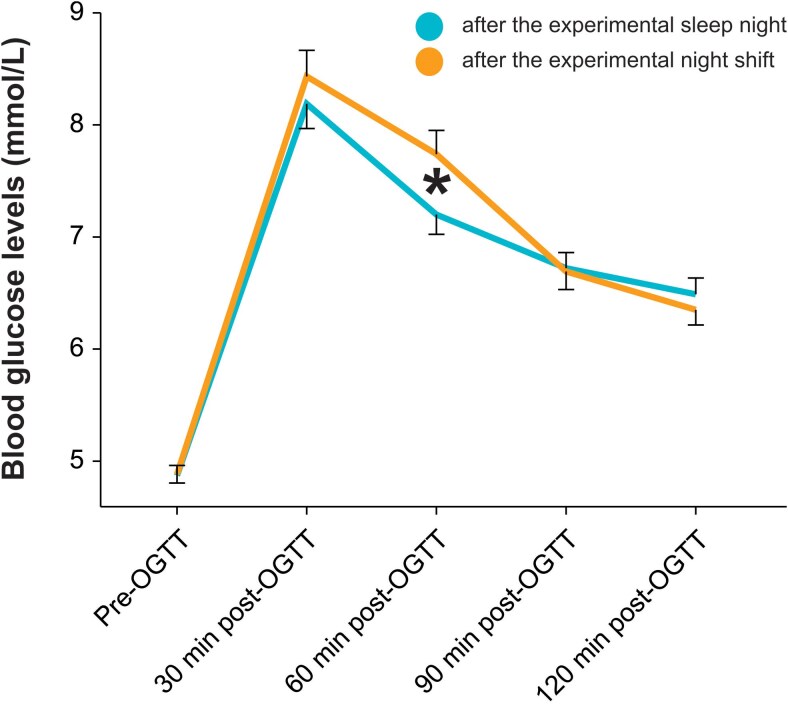
Mean (±SEM) blood glucose trajectories before and after an OGTT, separated by experimental condition. A significant interaction between condition and time was observed, and post-hoc comparisons were performed for each post-OGTT time point. Asterisk (*) denotes a statistically significant difference (*P* < .05) between conditions, derived from a linear mixed-effects model including subject as a random factor, experimental condition as a categorical factor, and baseline (pre-OGTT) blood glucose as well as progesterone-to-estradiol ratio (measured before each OGTT in the respective experimental condition) as independent variables.

The time course of blood glucose during the OGTT, stratified by condition, is shown in Fig. 2. A significant condition × time point interaction was detected (F(3, 48.83) = 3.19, *P* = .032). Simple-effects analyses revealed a significant difference at 60 minutes post-OGTT, with higher blood glucose levels following the experimental night shift condition (adjusted estimated mean difference vs sleep = 0.58 mmol/L, 95% CI: 0.11 to 1.05, *P* = .016). No other time points showed significant between-condition differences (*P* ≥ .143; Fig. 2). In addition, neither the condition × P/E ratio nor the P/E ratio × time point interaction reached significance (*P* ≥ .220).

**Figure 3:**
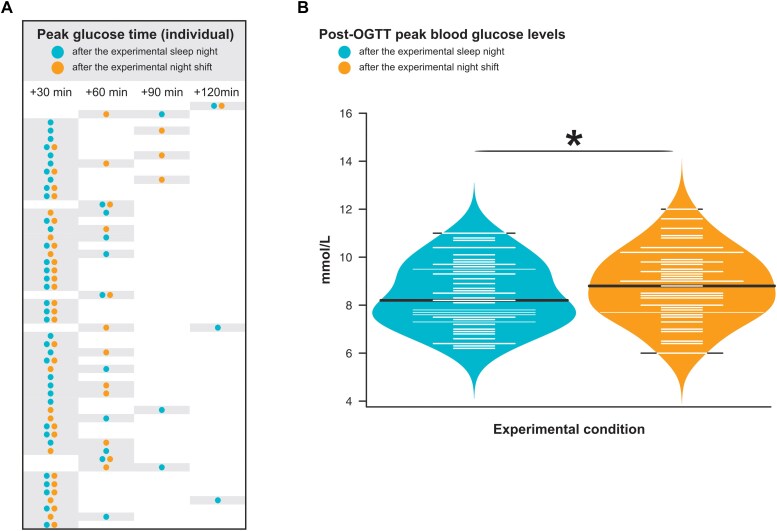
Effect of sleep vs night shift on post-ogtt peak glucose timing and levels. (A) Individual post-OGTT peak blood glucose times for each participant. Each dot represents the peak blood glucose time for a single individual, measured either after the experimental sleep night or after the experimental night shift. (B) Post-OGTT peak blood glucose levels shown as a bean plot, split by experimental condition. The width of each “bean” reflects the distribution of glucose values. White horizontal lines within the beans represent individual participants’ peak blood glucose values. The central thick black line represents the median for each condition. Asterisk (*) denotes a statistically significant difference (*P* < .05) between conditions, derived from a linear mixed-effects model including subject as a random factor, experimental condition as a categorical factor, and baseline (pre-OGTT) blood glucose as well as progesterone-to-estradiol ratio (measured before each OGTT in the respective experimental condition) as covariates.

Analysis of individual participants’ postprandial peak blood glucose responses showed that the time of the peak did not differ between conditions (χ² test, *P* = .850; Fig. 3A). The condition × P/E ratio interaction was not significant (*P* = .059). In the main-effects model, peak blood postprandial glucose was significantly higher following the experimental night shift condition compared with sleep (adjusted mean difference = 0.42 mmol/L, 95% CI: 0.03 to 0.81, *P* = .037; Fig. 3B). The P/E ratio was not associated with peak blood glucose levels (β = 0.12 [−0.40 to 0.64], *P* = .640).

### Sensitivity analysis

As summarized in Table 1, pre-OGTT RQ values were slightly but significantly lower after the experimental night shift condition compared with the sleep condition (adjusted mean difference = −0.029, 95% CI: −0.044 to −0.014, *P* < .001), indicating greater reliance on non-carbohydrate substrates following the night shift. No significant differences in RQ were observed during the postprandial period (Table 1).

**Table 1:** Pre- and post-OGTT RQ estimates, separated by experimental condition.

Time point*^a^*	Experimental condition, β [95% CI]		*P*-value
	*Night shift*	*Sleep*	
Pre-OGTT	0.756 [0.741 to 0.772]	0.785 [0.766 to 0.804]	<.001
+30 minutes	0.792 [0.775 to 0.808]	0.808 [0.788 to 0.828]	.076
+60 minutes	0.820 [0.800 to 0.840]	0.817 [0.796 to 0.838]	.799
+90 minutes	0.829 [0.802 to 0.838]	0.819 [0.797 to 0.842]	.929
+120 minutes	0.819 [0.799 to 0.839]	0.817 [0.795 to 0.839]	.786

Abbreviations: OGTT, oral glucose tolerance test; RQ, respiratory quotient.

Parameter estimates (β) and their 95% Confidence interval (95%-CI) were derived from linear mixed-effects models that included subject as a random factor, the progesterone-to-estradiol (P/E) ratio as a covariate, and experimental condition and time points as categorical variables. A significant interaction between condition and time points was observed (F (4, 49.69) = 2.75, *P* = .038); therefore, the post hoc comparisons summarized in the table were conducted. No significant interactions were found between the P/E ratio and time point or between the P/E ratio and condition (*P* ≥ .338). Consequently, no post hoc comparisons were performed for these terms.

^*a*^Time relative to ingestion of the oral glucose solution.

To assess whether this significant difference in pre-OGTT RQ contributed to the observed 60-minute post-OGTT glucose differences between conditions (see above and Fig. 2), we fitted LMMs including subject as a random factor, experimental condition as a categorical factor, and the P/E ratio as a covariate, with additional adjustment for pre-OGTT RQ. After accounting for pre-OGTT RQ, the difference in 60-minute glucose between the experimental night shift and sleep conditions was no longer significant (β [95% CI], experimental night shift vs sleep: 7.67 [7.27 to 8.08] vs 7.27 [6.91 to 7.63] mmol/L, *P* = .097). A similar pattern was observed for peak postprandial blood glucose, which occurred on average at ∼40 minutes under both conditions (Fig. 3A). Adjustment for pre-OGTT RQ attenuated the difference in peak glucose between the night shift and sleep conditions (β [95% CI], 8.77 [8.38 to 9.17] vs 8.46 [8.10 to 8.83] mmol/L, *P* = .146).

## Discussion

The present study, involving a cohort of women of reproductive age with normal HbA1c, demonstrated that post-OGTT glucose surges measured in the morning after one night of wakefulness—mimicking a night shift scenario—were greater than those observed after a night of sleep. Our findings are consistent with prior studies in men, which reported higher post-OGTT glucose excursions following overnight wakefulness, without accompanying changes in insulin (18).

Previous studies in approximately 3000 participants have linked higher glucose peaks during an OGTT to increased arterial stiffness and maladaptive arterial remodeling (24). Furthermore, in a cohort of 2957 Japanese community-dwelling adults without diabetes (aged 40-79 years), elevated 30-minute post-OGTT glucose levels (cut-off 9.6 mmol/L) were associated with an increased risk of developing type 2 diabetes, further highlighting the prognostic significance of early post-OGTT glucose (25). In the present study, although 60-minute and peak (∼30-minute) post-OGTT glucose were statistically higher following a single night without sleep compared with a standard night of sleep, the absolute mean group values for both experimental conditions remained below the 9.6 mmol/L threshold linked to future type 2 diabetes risk (25). These findings indicate that one night of wakefulness produces only a modest, transient impairment in next-day glucose tolerance.

Of note, our study examined only the effects of one night without sleep, so the impact of consecutive night shifts on postprandial glucose remains uncertain. Nonetheless, repeated exposure to night shifts, as experienced by shift workers, may produce cumulative impairments in glucose metabolism, consistent with epidemiological evidence linking long-term night shift work to elevated risk of type 2 diabetes (2-5). Even modest acute increases in post-OGTT glucose could therefore become more clinically meaningful over multiple consecutive nights, supporting the continued consideration of meal timing and composition strategies for night shift workers.

Importantly, no condition-related differences were observed in LAR, a marker reflecting both fasting and postprandial insulin sensitivity (16), suggesting that impaired insulin-dependent glucose disposal is unlikely to fully explain the elevation in post-OGTT glucose observed after the experimental night shift. We note, however, that postprandial insulin was not measured, nor did we assess metabolism in tissues relevant for glucose disposal, such as skeletal muscle; therefore, contributions from altered insulin secretion or peripheral insulin resistance cannot be fully excluded. Nonetheless, additional factors, such as shifts in energy-substrate utilization, may contribute to the observed postprandial glucose response. In the experimental night shift condition, the pre-OGTT RQ measured via indirect calorimetry was slightly lower, indicating increased fat oxidation. A shift toward fat oxidation can suppress glucose oxidation after glucose ingestion, consistent with the substrate competition described by the glucose–fatty acid (Randle) cycle. When glucose becomes available, lipid-oxidative pathways do not immediately downregulate, delaying glucose oxidation and causing a transient postprandial increase in blood glucose (26).

A recent study suggested that eating at night in a night shift–like setting is associated with worse daytime glucose control, whereas restricting meals to daytime hours can mitigate these night shift–related effects on glucose regulation (27). In the present study, participants were kept fasted overnight in both experimental conditions, which is a strength because it eliminates the potential confounding effect of nighttime eating, making comparisons of next-morning glucose responses between the experimental sleep and night shift conditions more straightforward. We found that the following morning, a lower pre-OGTT RQ—indicative of greater non-glucose substrate oxidation (17)—partially accounted for post-OGTT glucose differences between the experimental night shift and sleep conditions. These findings raise the question of whether interventions aimed at increasing glycogen stores prior to a night shift, or slowing their depletion during the shift, could mitigate impairments in next-day postprandial glucose control—a hypothesis that warrants direct experimental testing.

Previous evidence suggests that higher progesterone levels—or menstrual cycle phases in which progesterone predominates—are associated with impaired glucose metabolism, whereas higher estradiol levels or estradiol-dominant phases are linked to improved glucose handling (28-31). In the present study, the P/E ratio was neither statistically associated with fasting nor postprandial metrics of glucose metabolism, nor did it significantly interact with the experimental condition for any of these measures. These findings suggest that a single night of wakefulness impairs morning glucose tolerance, largely independent of inter-individual variations in progesterone and estradiol. Further research incorporating intra-individual fluctuations in these female sex hormones and extended glucose monitoring is needed to clarify whether menstrual-cycle dynamics modulate vulnerability to night shift metabolic stress.

### Strengths and limitations

A major strength of this study is that, to our knowledge, it is the first to examine whether night shift–induced impairments in next-morning glucose metabolism are influenced by menstrual cycle hormones. This is particularly relevant for occupational health, as many reproductive-aged women work night shifts in professions such as nursing and geriatric care.

Notwithstanding these strengths, several limitations should be considered. We focused on inter-individual differences in the P/E ratio, leaving intra-individual hormonal fluctuations across the menstrual cycle unexplored. Menstrual cycle phase was not included as a separate covariate, as the P/E ratio reflects the hormonal milieu at the time of testing; however, we acknowledge that phase-specific effects could influence metabolic responses.

The OGTT was conducted in the morning after an overnight fast, consistent with standard clinical protocols; however, fasting and postprandial glucose responses may vary across the day (32). Future studies combining standardized meals with continuous glucose monitoring could clarify potential diurnal effects and interactions with menstrual-cycle hormones.

All participants completed the regular sleep condition first, followed by the night shift condition, to mimic the real-world transition from a standard sleep period to a single night of night shift work. Procedural familiarity or reduced anxiety on the second visit could influence glucose outcomes. While procedural familiarity is likely of lesser concern, as suggested by previous studies showing that repeated OGTTs provide consistent estimates of glucose and insulin responses across consecutive days (33), familiarity with the protocol could still have reduced psychological stress (not measured herein) and influenced the results, as acute stress has been shown to elevate postprandial glucose (34). Consequently, the observed elevations in morning post-OGTT glucose following the night-shift condition, which was scheduled second, may actually underestimate the true effect that would be observed if the conditions had been fully counterbalanced.

Postprandial insulin was not measured; however, the absence of differences in LAR suggests that the observed post-OGTT glucose elevations were not driven solely by insulin-related changes. Future studies should directly measure postprandial insulin and other glucoregulatory hormones (eg, glucagon and GLP-1) to more comprehensively characterize the mechanisms underlying night shift–related alterations in glucose metabolism.

Given the modest sample size, the possibility of a Type II error cannot be fully excluded. Nevertheless, most previously published experimental studies examining the metabolic effects of acute circadian misalignment have included between 9 and 26 participants (6-10). In this context, our study—with 52 participants completing the sleep condition and 49 completing the night shift condition—represents one of the largest controlled experimental investigations in this field to date. Future studies with larger samples and repeated testing across menstrual phases will be required to validate and extend our findings.

## Conclusions

One night of wakefulness, resembling a night shift scenario, impairs next-morning glucose tolerance in women. These findings suggest that modifying the initial meal consumed after a night shift—for example, by selecting low-carbohydrate or high-fibre, low–glycemic index foods—may help reduce the risk of postprandial glucose excursions.

## Data Availability

Data presented herein are available as deidentified datasets upon request to researchers at other institutions, contingent upon signing a data access agreement prior to release. For inquiries, please contact Pei Xue (pei.xue@uu.se).

## Abbreviations

HOMA-IR: homeostatic model assessment of insulin resistance
LAR: leptin-to-adiponectin ratio
LMMs: linear mixed-effects models
OGTT: oral glucose tolerance test
P/E ratio: progesterone-to-estradiol ratio
RQ: respiratory quotient
VCO_2_: carbon dioxide production
VO_2_: oxygen consumption
